# Characterisation of interrelations among myocardial work parameters in patients with hypertrophic cardiomyopathy: differences between non-obstructive and obstructive disease

**DOI:** 10.3389/fcvm.2025.1670503

**Published:** 2026-02-11

**Authors:** Viktória Nagy, Gergely Rácz, Krisztina Boda, Hedvig Takács, Bianka Polestyuk, Noémi Schvartz, László Dániel Vidács, Jenő Antal Pintér, Attila Pálinkás, Árpád Kormányos, Tamás Szűcsborus, János Borbás, Tamás Szili-Török, Róbert Sepp

**Affiliations:** 1 Division of Non-Invasive Cardiology, Cardiology Centre, Department of Internal Medicine, University of Szeged, Szeged, Hungary; 2 Department of Medical Physics and Informatics, University of Szeged, Szeged, Hungary; 3 Department of Medicine, Elisabeth Hospital, Hódmezővásárhely, Hungary

**Keywords:** advanced echocardiography, global longitudinal strain (GLS), hypertrophic cardiomyopathy, left ventricular outflow tract obstruction, myocardial work

## Abstract

**Background/objectives:**

In recent years, novel echocardiographic parameters, known as myocardial work (MW) parameters, have been introduced into clinical practice for the assessment of contractile function. This study aimed to provide a technical characterization of the interrelations among these MW parameters and evaluate their differences between patients with non-obstructive (nHCM) and obstructive hypertrophic cardiomyopathy (oHCM).

**Patients and methods:**

One-hundred-eighteen patients with HCM, including 68 nHCM and 50 oHCM patients were assessed. Global longitudinal strain (GLS) and derived global MW parameters—including global work index (GWI), global constructive work (GCW), global wasted work (GWW), and global work efficiency (GWE)—were determined by 2D-speckle tracking echocardiography.

**Results:**

In all HCM cohorts, GLS demonstrated a strong, significant correlation with GWI and GCW (*r*: −0.619 to −0.818), whereas the correlation between GLS and GWW was considerably weaker (*r*: 0.320 to 0.373), consistent in both univariate correlation and multiple regression analyses. A strong, significant correlation was observed between GWI and GCW, and a significant correlation existed between GWW and GWE. Within HCM subgroups characterized by increasing left ventricular outflow tract (LVOT) gradients, GWI/GCW values exhibited “pseudonormalization” in the obstructive HCM groups, due to the offsetting effects of the nominal decrease in GLS and the nominal increase in LVOT gradient. In contrast, GWW values increased gradually with increasing LVOT gradients, and the difference compared to controls was significant even in the HCM group with LVOT gradients <10 mmHg.

**Conclusions:**

Given the strong correlation between GLS and GWI/GCW, it is probable that changes in GLS would result in corresponding changes in GWI/GCW, potentially limiting the incremental discriminatory value of these parameters beyond GLS. GWW appears to be the most independent MW parameter from GLS in patients with hypertrophic cardiomyopathy as it weakly correlates with GLS, unlike GWI/GCW.

## 1 Introduction

Hypertrophic cardiomyopathy (HCM) is the most common heritable heart muscle disease, characterized by sarcomeric gene mutations that lead to profound alterations in myocardial contraction and relaxation (1, 2). At the cellular level, pathophysiological hallmarks of the disease include hypercontractility, impaired relaxation, increased energy consumption and myocardial wall stress, which are caused by extra cross-bridge formation and dysregulation of the super-relaxed state of myosin heads (3, 4). These cellular abnormalities result in organ-level morphological and functional alterations, including myocardial hypertrophy, left ventricular outflow tract (LVOT) obstruction, diastolic dysfunction, small vessel disease, and myocardial ischemia.

As HCM is fundamentally a disease of myocardial hypercontractility, a method that provides direct information about contractility is an investigational method of primary importance. The recent approval of direct myosin inhibitors (5) to treat obstructive HCM (6, 7)—drugs that specifically target contractility—further elevates the need for such a methodological assessment. Speckle tracking echocardiography is increasingly applied in the field of cardiomyopathies, not only for evaluating ventricular function but also for assessing atrial function (8). In recent years, novel echocardiographic parameters, known as myocardial work (MW) parameters, have been introduced into clinical practice for the assessment of contractile function (9, 10). These parameters constitute a group of novel parameters that utilize pressure-strain loops to estimate myocardial performance. The method has been validated and has shown excellent agreement with the invasive pressure-volume loop, a well-established method of left ventricular (LV) functional assessment (11). The basics and the clinical applications of MW, including hypertension, coronary artery disease, heart failure, cardiac resynchronization therapy, HCM and athlete's heart, amyloidosis, dilated cardiomyopathy, valvular heart disease and cardio-oncology, has recently been reviewed by Trimarchi et al. (10).

Given that MW parameters are derived from global longitudinal strain (GLS) and are intended to provide load-independent measurements, it is crucial to assess whether MW parameters offer any incremental value over GLS in the various aspects of HCM assessment. To reliably establish the value of MW parameters in HCM, we must fully characterize the relationship between GLS and MW parameters, including their association with the disease's morphological and clinical manifestations, severity, and outcome. However, the pathophysiology of HCM is highly complex, involving primary determinants of contractility (e.g., sarcomeric gene mutations, myofiber disarray, fibrosis, myocardial ischemia, etc.) and secondary determinants (e.g., obstruction, diastolic dysfunction, etc.). This complexity necessitates a comprehensive, multi-faceted approach to addressing this condition.

Assessment of MW parameters in HCM, beyond describing basic changes related to MW parameters in non-obstructive HCM (nHCM) (12–15), have mainly focused on special subsets of HCM (e.g., apical HCM) (16), evaluated differences between nHCM and phenocopies [e.g., ATTR amyloidosis (17–19), athlete's heart (20)], or investigated specific issues related to the topic [relation of MW parameters to exercise tolerance (21), perfusion defects (22), or myocardial fibrosis (23, 24)]. On the other hand, MW parameters have been rarely evaluated in patients with obstructive HCM (oHCM), and the number of oHCM patients assessed in these studies has been surprisingly low, including only 5–21 oHCM patients (20, 22, 23). Moreover, MW parameters themselves have never been directly compared between nHCM and oHCM patients. One of the reasons for this is that the non-invasive estimation of LV systolic peak pressure in oHCM, and therefore the possibility to calculate MW in oHCM, has only recently been validated (25). As a consequence, in previous studies on oHCM patients, the left ventricular pressure (LVP), one of the determinants of MW parameters, was calculated incorrectly by simply adding the resting peak LVOT gradient to the systolic blood pressure (SBP) (20, 22, 23), which overestimates LVP, and therefore, MW parameter values (25).

MW parameters are derived from global longitudinal strain (GLS) and LVP, which are in turn calculated from SBP and LVOT resting and average gradients. Given that the precise algorithm for MW parameter calculation is not publicly available, characterizing the interrelationships between MW parameters and their constituent components is essential. This characterization allows for a determination of whether changes in MW parameters reflect primary cardiac alterations or are simply a consequence of changes in their independent components (GLS, SBP, LVOT gradient). Furthermore, understanding the differences in MW parameters between obstructive HCM (oHCM) and non-obstructive HCM (nHCM) is crucial, as the LVOT gradient, a defining feature of oHCM, directly influences LVP. Therefore, this study aimed to delineate the interrelationships among MW parameters and their constituent components, and to compare MW parameters between oHCM and nHCM patients. This manuscript reports the results of a technical analysis to characterize the relationship between GLS and MW parameters, focusing on their interrelationships and differences between the obstructive and non-obstructive subgroups, thereby providing essential insights into their interaction.

## 2 Patients and methods

### 2.1 Patients

Consecutive patients with a proven diagnosis of HCM were enrolled into the study. Diagnosis of HCM was established based on published diagnostic criteria (1). Patients with a confirmed diagnosis of a HCM phenocopy (e.g., Fabry disease, ATTR amyloidosis, mitochondrial cardiomyopathy, etc.) and with suboptimal echocardiographic images were excluded. Although LVOT gradient is a continuous variable and, as such, affects MW parameters in a continuous manner, following clinical convention, we defined a non-obstructive HCM (nHCM, patients with <30 mmHg LVOT gradient) and an obstructive (oHCM, patients with >30 mmHg LVOT gradient) HCM group in addition to the total HCM group. To better characterize the differences in relation to MW parameters between controls and non-obstructive and obstructive HCM categories, different HCM subgroups of increasing LVOT gradients (<10 mmHg, <30 mmHg, >30 mmHg, >50 mmHg, >70 mmHg) were created. For comparing MW parameters and component parameters of MW to different subgroups of patients with HCM, an age- and sex-matched group of ostensibly healthy subjects served as a control group. None of the control group subjects had a known history of any illness, whether cardiovascular or non-cardiovascular, nor were they taking any medication.

The investigation conforms with the principles outlined in the Declaration of Helsinki (Br Med J 1964; ii: 177). The study was approved by the Hungarian Medical Research Council (8489-2/2018/EÜIG, dated February 16, 2018; 08783-2/2023/EÜIG, dated March 7, 2023; 628-1/2018/EKU ETT TUKEB) and the Institutional Research Ethics Committee of the University of Szeged (148/2024-SZTE IKEB, dated November 11; 2024). All subjects participating in the study gave prior written informed consent to participate in the study.

### 2.2 Methods

**Figure 1:**
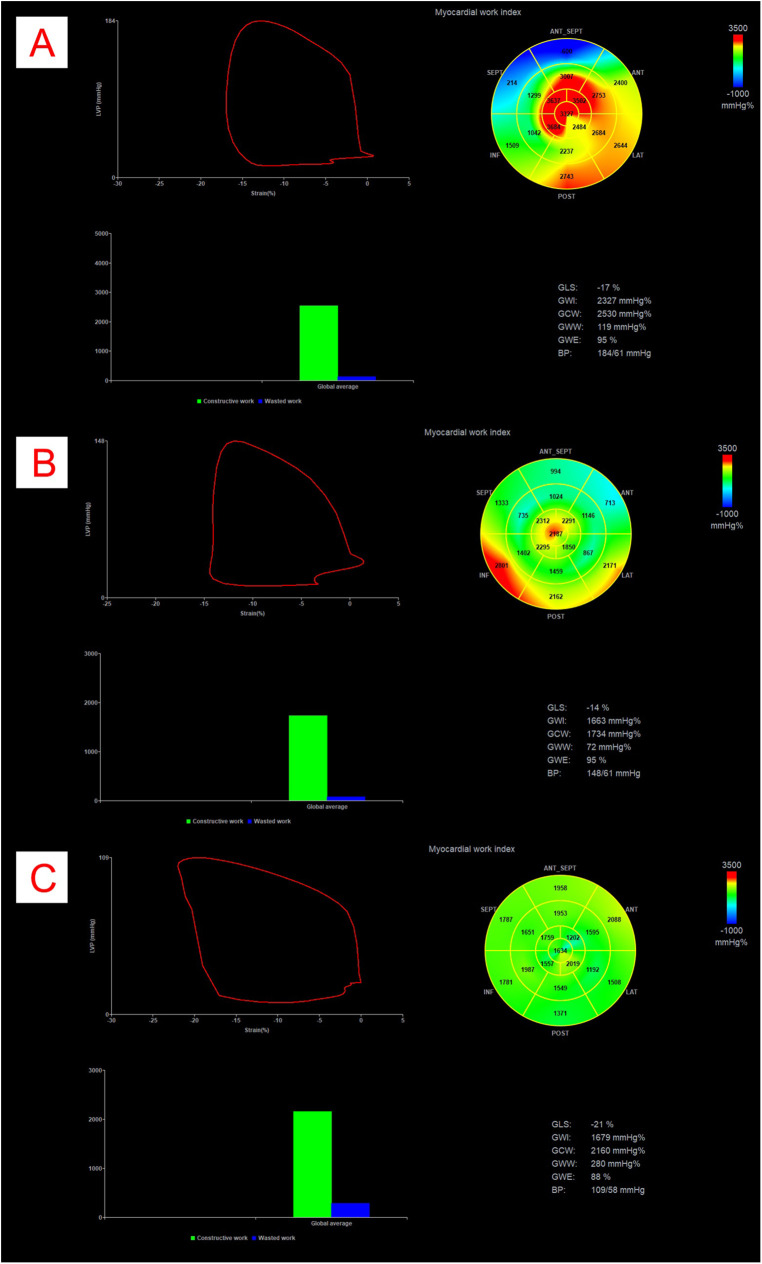
Example of myocardial work analysis in a patient with obstructive HCM **(Panel A)**, with non-obstructive HCM **(Panel B)** and in a control patient **(Panel C)**. All panels represent pressure-strain loops, bullseye maps showing segmental GWI values, bar graph representations of GCW vs. GWW, and numerical values for GLS, GWI, GCW, GWW, GWE and BP. The area within the loop represents GWI. BP, blood pressure; GCW, global constructive work; GLS, global longitudinal strain; GWI, global work index; GWW, global wasted work.

In addition to recording the main demographic, clinical and laboratory parameters, complete standard and 2D-speckle tracking echocardiographic examination was performed in patients. Resting blood pressure was measured in the supine position immediately before the echocardiographic examination. All patients underwent comprehensive echocardiography, including 2D speckle tracking echocardiography of the LV, right ventricle, and left atrium, as well as non-invasive MW analysis. All measurements were ECG-assisted or -gated. Standard cardiac dimensions were obtained and indexed to body surface area (BSA) where appropriate. LV systolic function was assessed comprehensively, including ejection fraction measurement using the biplane Simpson's method. LVOT gradient was assessed at rest and during the Valsalva manoeuvre to determine peak instantaneous and provoked gradients, respectively. Echocardiographers carefully adjusted the Doppler angle from the left atrium to the LVOT to differentiate mitral regurgitation from LVOT flow. Diastolic function was evaluated according to current guidelines, incorporating tissue velocity imaging (TVI). Left heart speckle-tracking strain analysis included GLS measurement from apical 2-, 3-, and 4-chamber views. From these data, the following global MW parameters were derived: global work index (GWI), global constructive work (GCW), global wasted work (GWW), and global work efficiency (GWE) (Figure 1). LV peak pressure, used in the GWI, GCW, GWW, and GWE calculations, was determined as previously described (25). All examinations were carried out with a GE Vivid E95 R4 (GE Healthcare, Horten, Norway) cardiac ultrasound system.

### 2.3 Statistical analysis

For descriptive statistics, continuous variables were expressed as mean ± standard deviation (SD) or median (interquartile range, IQR), and categorical variables were given as the number of cases (%). The Kolmogorov–Smirnov and the Shapiro–Wilks tests were used to test the normality of the distribution of continuous variables. Chi-square tests were used for categorical variables. Spearman's correlation analysis was used to analyse correlations between continuous variables. Multiple linear regression analyses were performed to examine the relationship between dependent MW parameters and independent components of MW parameters. GWI, GCW, GWW and GWE were considered as dependent parameters, while GLS, SBP, LVOT resting peak gradient values were considered as independent parameters (referred to as “component” or “constituent” parameters in this text). All independent variables were entered into the model with the “Enter” method in a single step. Differences between controls and HCM subgroups were analyzed by Student's t-test assuming unequal variances. To reduce the risk of Type I error increase, *p*-values for the comparisons among multiple groups were corrected by FDR throughout the paper. As for determining the abnormal range for the MW parameters, data from the EACVI NORRE study were used, as follows (normal range, or upper/lower limit of normal): GWI: 1,295–2,505 mmHg%, GCW: 1,582–2,881 mmHg%, GWW: 226 mmHg%, GWE: 91% (26).

Statistical analysis was conducted with MedCalc® Statistical Software version 20.106 (MedCalc Software Ltd., Ostend, Belgium; https://www.medcalc.org; 2022) and IBM SPSS for Windows version 29. FDR corrections of the unique *p*-values was performed with R. A *p* < 0.05 value was considered as statistically significant.

## 3 Results

### 3.1 Patient group characteristics

One-hundred-eighteen patients with HCM [78 men (66%), mean age: 53 ± 13 years] were included into the study. The clinical, demographic and echocardiographic characteristics of the patients are presented in Table 1. Among the HCM patients there were 68 patients [46 (67%) men, mean age: 53 ± 12 years] with nHCM and 50 patients [32 (63%) men, mean age: 53 ± 13 years] with oHCM. The control group comprised 32 ostensibly healthy subjects [15 (47%) males, mean age: 47 ± 9 years].

**Table 1:** Demographic, clinical and echocardiographic parameters of the study population.

Variable	Control group (*n* = 32)	Total HCM group (*n* = 118)		nHCM < 30 group (*n* = 68)	oHCM > 30 group (*n* = 50)	
	mean ± SD/median (IQR)	mean ± SD/median (IQR)	*p* *	mean ± SD/median (IQR)	mean ± SD/median (IQR)	*p* ^†^
Demographic and clinical parameters						
Age (years)	47 ± 9	53 ± 13	0.060	53 ± 12	53 ± 13	0.869
Male sex, *n* (%)	15 (47%)	78 (66%)	0.071	46 (67%)	32 (63%)	0.786
BSA (m^2^)	2 (1.8–2.1)	2 (1.9–2.2)	0.078	1.9 (1.9–2.2)	2.1 (1.9–2.2)	0.452
Familial history of HCM, *n* (%)	─	31 (26%)	─	16 (23%)	15 (29%)	0.623
Coronary artery disease, *n* (%)	**0** **(0%)**	**15** **(13%)**	**<0.0001**	**4** **(6%)**	**11** **(21%)**	**0** **.** **025**
Diabetes mellitus, *n* (%)	0 (0%)	12 (10%)	0.061	9 (13%)	3 (6%)	0.522
Hypertension, *n* (%)	**4** **(13%)**	**65** **(55%)**	**<0.0001**	40 (59%)	25 (50%)	0.627
Atrial fibrillation, *n* (%)	**0** **(0%)**	**22** **(19%)**	**<0.0001**	16 (24%)	6 (12%)	0.176
Heart rate (bpm)	68 (64–78)	66 (60–74)	0.243	66 (60–74)	66 (60–72)	0.760
Systolic blood pressure (mmHg)	134 (123–140)	132 (119–146)	0.675	130 (118–140)	138 (124–150)	0.085
Diastolic blood pressure (mmHg)	77 (70–83)	74 (64–83)	0.443	74 (65–82)	74 (63–83)	0.869
NYHA class (>2), *n* (%)	─	18 (15%)	─	9 (13%)	9 (18%)	0.310
QRS (ms)	─	107 (97–122)	─	105 (94–122)	111 (100–130)	0.295
NTproBNP (pg/mL)	─	717 (327–1,752)	─	**635** **(237–1,136)**	**823** **(456–2,192)**	**0** **.** **025**
Troponin (ng/l)	─	16 (11–27)	─	15 (10–25)	17 (12–32)	0.138
Pace-maker implantation, *n* (%)	─	6 (5%)	─	3 (4%)	3 (6%)	0.635
ICD implantation, *n* (%)	─	28 (24%)	─	19 (28%)	9 (18%)	0.130
ESC SCD score (%)	─	3.7 (2.8–6.4)	─	**3.2** **(2.6–5.4)**	**5.2** **(3.6–6.9)**	**0** **.** **020**
Echocardiographic parameters						
Left atrial volume, LAV (mL)	**51** **(47–60)**	**107** **(80–130)**	**<0.0001**	**100** **(72–125)**	**117** **(96–130)**	**0** **.** **023**
Left atrial volume index, LAVI (mL/m^2^)	**26** **(25–31)**	**53** **(41–68)**	**<0.0001**	**49** **(37–64)**	**56** **(47–71)**	**0** **.** **006**
End diastolic diameter, EDD (mm)	46 (41–50)	46 (42–50)	0.266	47 (42–50)	45 (43–50)	0.629
End diastolic volume, EDV (mL)	84.5 (71–106)	94 (76–114)	0.116	91 (72–115)	96 (82–112)	0.293
End systolic diameter, ESD (mm)	30 (27–33)	29 (26–33)	0.948	30 (26–34)	29 (26–32)	0.490
End systolic volume, ESV (mL)	32 (23–36)	30.5 (24–42)	0.261	32 (25–44)	30 (24–42)	0.560
Ejection fraction, EF (%)	69 (65–71)	66 (60–71)	0.704	65 (60–70)	68 (62–73)	0.054
Transmitral E wave velocity (cm/s)	77 (70–88)	78 (63–94)	0.826	**70** **(59–89)**	**88** **(71–104)**	**0** **.** **003**
Transmitral A wave velocity (cm/s)	61 (56–72)	76 (58–89)	0.074	**69** **(52–83)**	**82** **(74–98)**	**0** **.** **001**
E/A	1.3 (1.1–1.5)	1.1 (0.8–1.4)	0.110	1.1 (0.7–1.5)	1.1 (0.8–1.4)	0.547
Mitral annulus a’ velocity (cm/s)	**12** **(9–13)**	**9** **(7–11)**	**0.005**	8 (7–12)	9 (8–11)	0.678
Mitral annulus e’ velocity (cm/s)	**14** **(10–16)**	**8** **(6–10)**	**<0.0001**	8 (6–10)	7 (5–9)	0.161
E/e’	**5.8** **(4.9–7.7)**	**10** **(8–14)**	**<0.0001**	**9.3** **(7.4–11.8)**	**11.0** **(8.9–16.2)**	**0** **.** **005**
Maximal left ventricular wall thickness (mm)	─	23 (20–27)	─	22 (19–26)	24 (21–27)	0.077
Resting peak LVOT gradient (mmHg)	─	14 (0–68)	─	**0** **(0–12)**	**73** **(52–96)**	**<0** **.** **0001**
Valsalva peak LVOT gradient (mmHg)	─	27 (0–91)	─	**0** **(0–18)**	**110** **(79–140)**	**<0** **.** **0001**
TAPSE (mm)	24 (23–26)	22 (20–25)	0.137	22 (19–25)	22 (20–25)	0.591
Pulmonary pressure (mmHg)	**23** **(22–27)**	**32** **(25–40)**	**0.002**	30 24–38)	32 (28–47)	0.146

HCM, hypertrophic cardiomyopathy; nHCM, non-obstructive HCM; oHCM, obstructive HCM (for definition please see text); BSA, body surface area; NYHA, New York Heart Association class; QRS, QRS complex of the ECG; NTpBNP, N-terminal brain natriuretic peptide; ICD, implantable cardioverter defibrillator; ESC, European Society of Cardiology; SCD, sudden cardiac death; LVOT, left ventricular outflow tract; TAPSE, tricuspid annular plane systolic excursion.

Values are given as mean ± standard deviation (SD), median (interquartile range: IQR) or *n* (%). Significant differences are printed in bold.

* *p* is the *p* value between controls and the total HCM group, while

† *p* is the *p* value between patients in the non-obstructive (nHCM < 30) and the obstructive (oHCM > 30) HCM groups.

Age and sex distribution didn't differ between the groups. As the control group was free of any known major diseases (with the exception of hypertension which was present in 13% of the controls), many comorbidities were more frequent in the total HCM group (the presence of coronary artery disease, hypertension, atrial fibrillation) and many echocardiographic parameters were different in the total HCM group compared to the control group. The presence of coronary artery disease was slightly more frequent in the oHCM group, and NTproBNP levels and the ESC SCD score (1) were higher (presumably due to the presence of obstruction, a component parameter of the ESC SCD score). Some echocardiographic parameters of diastolic dysfunction were worse in oHCM patients (LAVI, transmitral A and E velocities), however, sensitive TDI parameters of diastolic function (mitral annulus a' and e' velocities) were not different, and E/e' was comparable between the two groups. LVOT gradients were obviously higher in the oHCM group.

### 3.2 Correlation of MW parameters (GWI, GCW, GWW and GWE) with component parameters of MW (GLS, LVP, SBP, resting peak and average LVOT gradient) in the total, non-obstructive and obstructive HCM cohorts

Given that MW parameters are derived from GLS and LVP (calculated from systolic BP, resting, and average LVOT gradient), we initially assessed the univariate correlations between MW parameters and their measured constituent parameters to identify the primary determinants of MW parameters (Table 2). GLS showed a very strong correlation with GWI and GCW (with *r* values of −0.619 to −0.818) in all HCM cohorts and the level of correlation was much stronger than that of LVP (with *r* values of 0.215 to 0.590) or any of the constituent parameters of LVP. The correlation between GLS and GWW was much weaker (with *r* values of 0.320 to 0.373) and in the nHCM group, the correlation with LVP or SBP was even stronger (with *r* values of 0.452 and 0.401) than that of GLS (*r* = 0.320). The correlation of LVP with MW parameters was usually stronger than with the individual components of LVP (systolic BP, resting peak and average LVOT gradient). It is of note that in the nHCM and oHCM groups SBP showed stronger correlation with MW parameters than peak resting LVOT gradient, which showed no significant correlation. The correlation between GWE (the derived parameter of GCW and GWW) and GLS was also strong, although weaker than that of GWI or GCW.

**Table 2:** Univariate correlation analysis of MW parameters with GLS, LVP, systolic BP, resting peak and average LVOT gradient in the total, non-obstructive (nHCM < 30) and obstructive (oHCM > 30) HCM cohorts.

Variable	Total HCM group (*n* = 118)				nHCM < 30 group (*n* = 68)				oHCM > 30 group (*n* = 50)			
	GWI	GCW	GWW	GWE	GWI	GCW	GWW	GWE	GWI	GCW	GWW	GWE
GLS (%)	−0.703****	**−0** **.** **619** ****	**0** **.** **330** ***	**−0** **.** **540** ****	**−0** **.** **797** ****	**−0** **.** **704** ****	**0** **.** **320** *	**0** **.** **484** ****	**−0** **.** **818** ****	**−0** **.** **712** ****	**0** **.** **373** *	**−0** **.** **644** ****
LVP	**0** **.** **500** ****	**0–590** ****	**0** **.** **547** ****	**−0** **.** **264** **	**0** **.** **334** *	**0** **.** **454** ***	**0** **.** **452** ***	**−**0.242	0.215	**0** **.** **347** *	0.285	−0.024
Systolic BP	**0** **.** **323** ***	**0** **.** **386** ****	**0** **.** **391** ****	**−**0.167	0.262	**0** **.** **395** **	**0** **.** **401** **	**−**0.215	0.246	**0** **.** **316** *	0.286	−0.026
Peak LVOTG	**0** **.** **445** ****	**0** **.** **490** ****	**0** **.** **407** ****	**−0.246** *	0.183	0.135	0.198	−0.224	0.170	0.302	0.124	0.065
Average LVOTG	**0** **.** **459** ****	**0** **.** **525** ****	**0** **.** **429** ****	**−0** **.** **250** *	0.241	0.264	0.235	−0.155	0.145	0.277	0.137	0.047

GLS, global longitudinal strain; LVP, left ventricular pressure; BP, blood pressure; LVOTG, left ventricular outflow tract gradient; GWI, global work index; GCW, global constructive work; GWW, global wasted work; GWE, global work efficiency.

Values represent Spearman's correlation coefficients (*r*).

Values are considered statistically significantly different at

* *p* < 0.05,

** *p* < 0.01,

*** *p* < 0.001,

**** *p* < 0.0001. Significant differences are marked with asterisks and printed in bold.

In multiple regression analyses (Table 3), similar to univariate correlation, partial correlation coefficients (partial *r* values) between GLS and GWI or GCW were notably high (ranging from −0.751 to −0.878), while those between GLS and GWW were much lower (0.154 to 0.284), not reaching significant levels. Systolic BP demonstrated strong partial correlations with GWI and GCW (0.614 to 0.850), although lower than those observed with GLS, except for GWW. The partial correlation between systolic BP and MW parameters were similar compared to resting LVOT peak gradients in the total and obstructive HCM groups. Within the nHCM group, systolic BP exhibited higher partial correlation with MW parameters compared to resting LVOT gradient. This is attributable to the fact that in this group, systolic BP is the primary determinant of LVP due to minimal or absent LVOT gradients. In contrast, for the oHCM group, partial correlations between MW parameters, systolic BP, and resting LVOT gradients were comparable (although not always significant), reflecting the closer proximity of LVOT gradient values to systolic BP values in this patient population.

**Table 3:** Multiple regression analysis of MW parameters with GLS, systolic BP, and resting peak LVOT gradient in the total, non-obstructive (nHCM < 30) and obstructive (oHCM > 30) HCM cohorts.

Variable	Total HCM group (*n* = 118)				nHCM < 30 group (*n* = 68)				oHCM > 30 group (*n* = 50)			
	GWI	GCW	GWW	GWE	GWI	GCW	GWW	GWE	GWI	GCW	GWW	GWE
GLS (%)	**−0.859** ****	**−0.820** ****	**0.220** *	**−0.303** **	**−0.857** ****	**−0.878** ****	0.154	−0.019	**−0.834** ****	**−0.751** ****	0.284	**−0.586** ****
Systolic RR (mmHg)	**0.692** ****	**0.734** ****	**0.353** ****	−0.102	**0.751** ****	**0.850** ****	**0.472** **	−0.256	**0.614** ****	**0.680** ****	**0.380** *	0.038
Peak LVOTG (mmHg)	**0.811** ****	**0.818** ****	**0.252** *	−0.041	0.257	0.200	0.133	−0.080	**0.603** ****	**0.594** ****	0.254	0.129
Coefficient of determination	**0.891** ****	**0.876** ****	**0.358** **	**0.347** **	**0.880** ****	**0.897** ****	**0.502** ***	**0.371** *	**0.884** ****	**0.848** ****	0.426	**0.629** ***

GLS, global longitudinal strain; BP, blood pressure; LVOTG, left ventricular outflow tract gradient; GWI, global work index; GCW, global constructive work; GWW, global wasted work; GWE, global work efficiency.

Values represent partial correlation coefficients, adjusted for age, sex, heart rate, LV ejection fraction, maximal LV wall thickness, E/e’, left atrial volume, presence of atrial fibrillation, coronary artery disease, and hypertension.

Values are considered statistically significantly different at

* *p* < 0.05,

** *p* < 0.01,

*** *p* < 0.001,

**** *p* < 0.0001. Significant differences are marked with asterisk and printed in bold.

### 3.3 Univariate correlation and multiple regression among MW parameters

In all HCM groups, a very strong correlation was observed between GWI and GCW (*r* values ranging from 0.910 to 0.948). Additionally, a strong correlation existed between GWW and GWE, with *r* values ranging from −0.672 to −0.746, in all HCM groups (Table 4).

**Table 4:** Univariate correlation analysis among MW parameters in the total, non-obstructive (nHCM < 30) and obstructive HCM (oHCM > 30) cohorts.

Variable	Total HCM group (*n* = 118)				nHCM < 30 group (*n* = 68)				oHCM > 30 group (*n* = 50)			
	GWI	GCW	GWW	GWE	GWI	GCW	GWW	GWE	GWI	GCW	GWW	GWE
GWI (mmHg%)	**1**				**1**				**1**			
GCW (mmHg%)	**0.948** ****	**1**			**0.915** ****	**1**			**0.910** ****	**1**		
GWW (mmHg%)	0.088	**0.259** **	**1**		−0.064	0.166	**1**		−0.096	0.102	**1**	
GWE (mmHg%)	**0.323** ***	0.187	**−0.718** ****	**1**	**0.382** **	0.201	**−0.746** ****	**1**	**0.552** ***	**0.422** **	**−0.672** ****	**1**

GWI, global work index; GCW, global constructive work; GWW, global wasted work; GWE, global work efficiency.

Values represent Spearman's correlation coefficients (*r*). Values are considered statistically significantly different at

* *p* < 0.05,

** *p* < 0.01,

*** *p* < 0.001,

**** *p* < 0.0001. Significant differences are marked with asterisks and printed in bold.

Multiple regression analyses confirmed the robust relationship between GWI and GCW, with partial correlation coefficients remaining high (0.911 to 0.964) across all HCM groups (Table 5). Similarly, the partial correlation between GWW and GWE remained strong, particularly in the oHCM group (partial *r*: −0.683).

**Table 5:** Multivariate regression analysis among MW parameters in the total, non-obstructive (nHCM < 30) and obstructive HCM (oHCM > 30) cohorts.

Variable	Total HCM group (*n* = 118)				nHCM < 30 group (*n* = 68)				oHCM > 30 group (*n* = 50)			
	GWI	GCW	GWW	GWE	GWI	GCW	GWW	GWE	GWI	GCW	GWW	GWE
GWI (mmHg%)	1				1				1			
GCW (mmHg%)	**0** **.** **964** ****	1			**0** **.** **964** ****	1			**0** **.** **911** ****	1		
GWW (mmHg%)	−0.531****	**0** **.** **603** ****	1		−0.644****	**0** **.** **667** ****	1		−0.316	**0** **.** **533** ***	1	
GWE (mmHg%)	0.083	0.020	−0.451****	1	0.049	0.004	−0.323**	1	0.146	0.170	−0.683****	1
Coefficient of determination	**0** **.** **941** ****	**0** **.** **944** ****	**0** **.** **557** ****	**0** **.** **373** ****	**0** **.** **933** ****	**0** **.** **933** ****	**0** **.** **553** ****	**0** **.** **226** ****	**0** **.** **922** ****	**0** **.** **923** ****	**0** **.** **649** ****	**0** **.** **716** ****

GLS, global longitudinal strain; GWI, global work index; GCW, global constructive work; GWW, global wasted work; GWE, global work efficiency.

Values represent partial correlation coefficients (*r*). Values are considered statistically significantly different at

* *p* < 0.05,

** *p* < 0.01,

*** *p* < 0.001,

**** *p* < 0.0001. Significant differences are marked with asterisks and printed in bold.

### 3.4 Comparison of GWI, GCW, GWW, GWE in HCM subgroups with different LVOT gradients with normal controls

Different HCM subgroups of increasing LVOT gradients were created (<10 mmHg, <30 mmHg, >30 mmHg, >50 mmHg, >70 mmHg) and GWI, GCW, GWW, GWE values were compared to that of normal controls (Table 6).

**Table 6:** Comparison of GLS, systolic BP, resting LVOT gradient and MW parameters between controls and different HCM subgroups of increasing LVOT gradients.

Variable	Control (*n* = 32)	Total HCM (*n* = 118)	nHCM, <10 (*n* = 46)	nHCM, <30 (*n* = 68)	oHCM, >30 (*n* = 50)	oHCM, >50 (*n* = 40)	oHCM, >70 (*n* = 28)
	mean ± SD	mean ± SD	mean ± SD	mean ± SD	mean ± SD	mean ± SD	mean ± SD
GLS (%)	−20.0 ± 1.5	−**15.1** **±** 4***	**−15.1** **±** 3.5****	**−15.5** **±** 3.6****	**−14.8** **±** 4.2****	**−14.7** **±** 4.3****	**−14.2** **±** 3.7****
systolic BP (mmHg)	132 ± 11	135 ± 21	135 ± 24	133 ± 22	138 ± 20	138 ± 22	141 ± 22
peak LVOTG (mmHg)	0 ± 0	**35** **±** 41***	**1** **±** 3*	**6** **±** 7****	**77** **±** 31****	**88** **±** 27****	**99** **±** 23****
GWI (mmHg%)	1,970 ± 218	1,693 ± 611	**1,443** **±** 462****	**1,482** **±** 473****	1,995 ± 636	2,067 ± 638	2,091 ± 654
GCW (mmHg%)	2,419 ± 233	2,146 ± 679	**1,828** **±** 501****	**1,883** **±** 522****	2,526 ± 683	2,620 ± 685	2,677 ± 704
GWW (mmHg%)	121 ± 82	**263** **±** 183***	**210** **±** 177**	**220** **±** 156***	**326** **±** 203****	**334** **±** 214****	**364** **±** 241****
GWE (mmHg%)	95 ± 3	**86** **±** 6***	**88** **±** 6****	**88** **±** 6****	**84** **±** 7****	**85** **±** 6****	**85** **±** 6****

GLS, global longitudinal strain; BP, blood pressure; LVOTG, left ventricular outflow tract gradient; GWI, global work index; GCW, global constructive work; GWW, global wasted work; GWE, global work efficiency; nHCM, non-obstructive HCM; oHCM, obstructive HCM; <10, <30, >30, >50, >70, left ventricular outflow tract gradient in mmHg, respectively.

Values are given as mean ± SD. Values are considered statistically significantly different at

* *p* < 0.05,

** *p* < 0.01,

*** *p* < 0.001,

**** *p* < 0.0001. Significant differences are marked with asterisk and printed in bold.

GLS values decreased gradually (from −15.1 to −14.2%) as LVOT gradients increased. This difference compared to controls was significant even in the HCM group with LVOT gradients <10 mmHg (−20.0 vs. −15.1%, *p* < 0.0001). Conversely, peak LVOT resting gradient values increased gradually (from 0 mmHg to 99 mmHg) with increasing LVOT gradient categories, and the difference compared to controls was significant in all HCM groups. Systolic BP was not different between the categories, so MW parameters were defined by almost entirely by the GLS and resting gradient.

**Figure 2:**
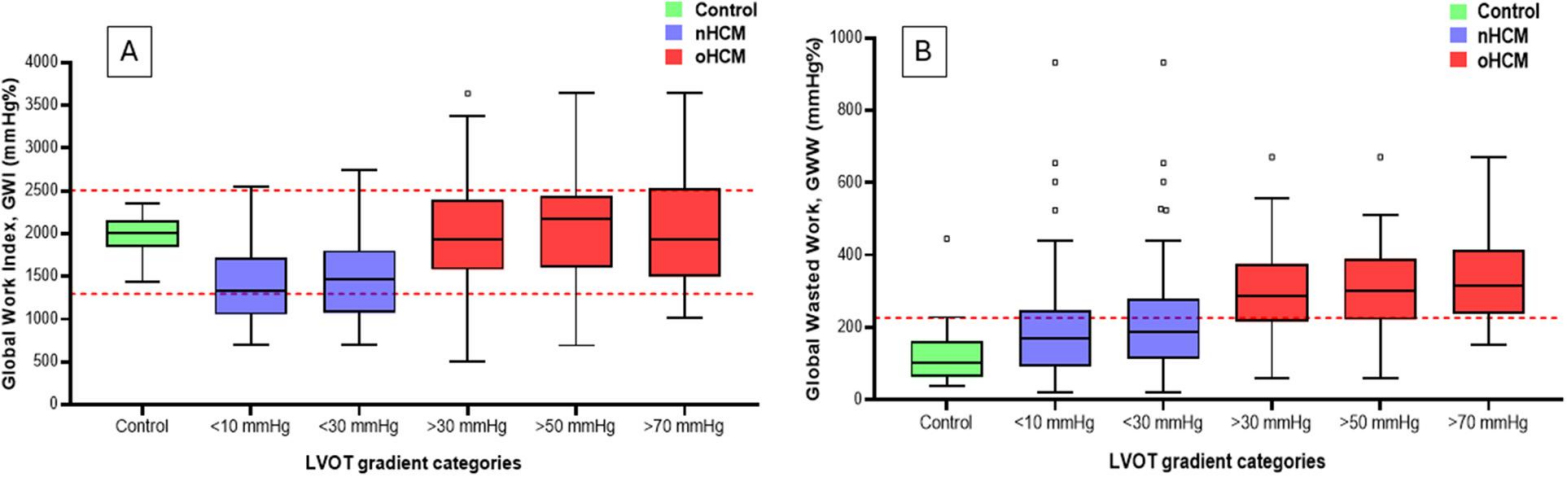
Graphical representation of changes in GWI **(Panel A)** and GWW **(Panel B)** among groups with different LVOT gradients and the control group. The dashed red lines represent the range for normal values for GWI and the upper limit of normal values for GWW. Note the “pseudonormalization” of GWI in the oHCM categories: in contrast to the nHCM categories, GWI values return to the normal range with increasing gradients, and are no longer different from that of the controls. GWW values rise gradually with increasing gradients, and are statistically different from that of controls even in the nHCM categories, and exceed the upper limit of normal in the oHCM categories. GWI, global work index; GWW, global wasted work; LVOT, left ventricular outflow tract; oHCM, obstructive hypertrophic cardiomyopathy; nHCM, non-obstructive hypertrophic cardiomyopathy.

Importantly, the difference between GWI or GCW values in controls and HCM subgroups with increasing LVOT gradients was significant only in the non-obstructive HCM group (with LVOT gradients <10 mmHg or <30 mmHg). In the obstructive HCM groups, this difference disappeared, even in the HCM group with LVOT gradients >30 mmHg. This was primarily due to the offsetting effects of the nominal decrease in GLS values and the nominal increase in resting LVOT gradient values (Figure 2). In contrast, GWW values increased gradually (from 210 to 364 mmHg%) with increasing LVOT gradients, and the difference compared to controls was significant even in the HCM group with LVOT gradients <10 mmHg (121 vs. 210 mmHg%, *p* = 0.0001) (Table 6 and Figure 2). Similar changes were observed regarding GWE values, exhibiting the opposite trend, decreasing gradually (from 88 to 85%) with rising LVOT gradients. The difference compared to controls was also significant even in the HCM group with LVOT gradients <10 mmHg (95 vs. 88%, *p* = 0.000).

**Figure 3:**
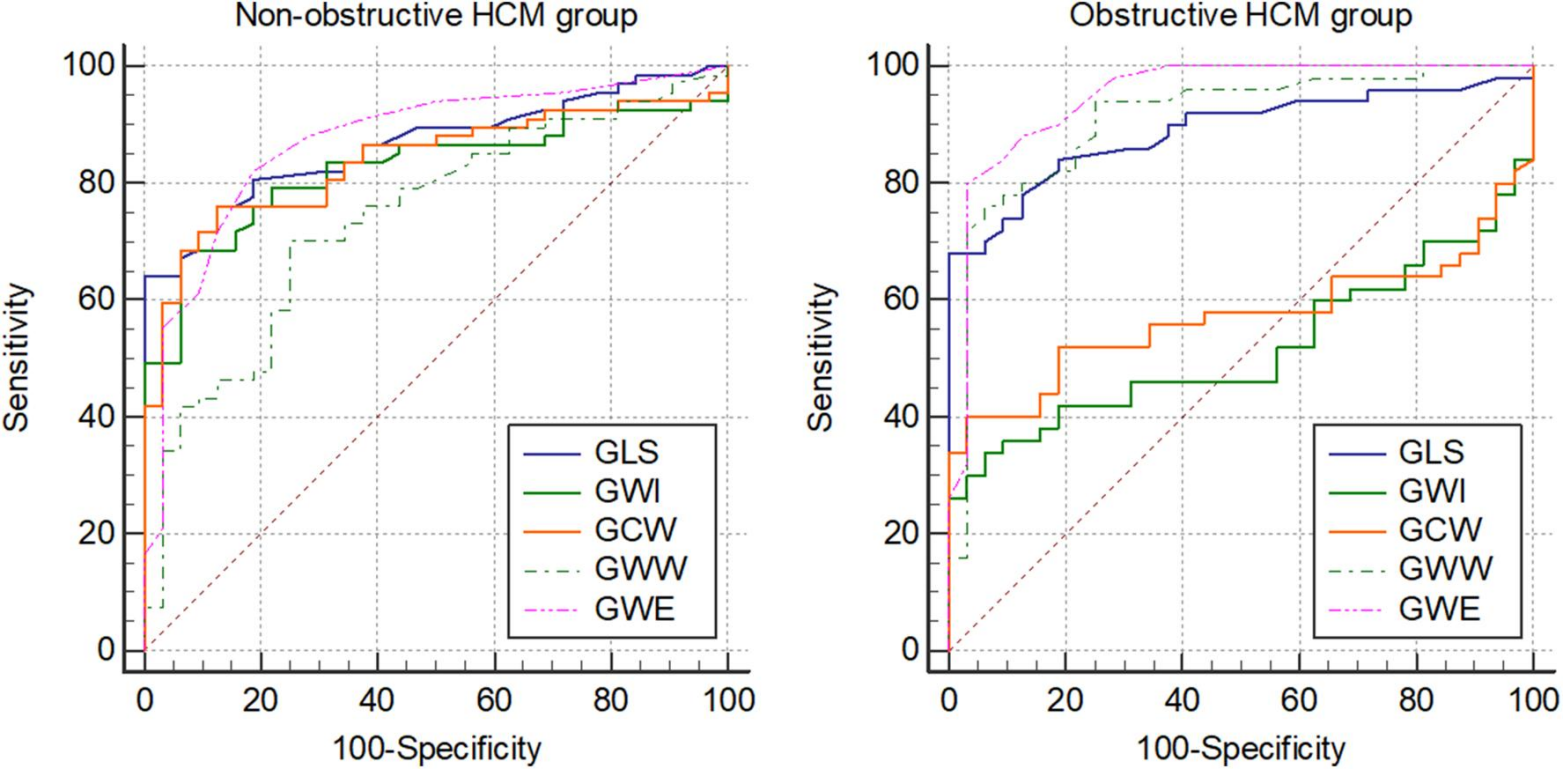
Graphical representation of ROC curve analysis of GLS and MW parameters between non-obstructive (resting peak LVOT gradient <30 mmHg) and obstructive (resting peak LVOT gradient >30 mmHg) HCM groups and the control group. ROC, receiver operator characteristics; GLS, global longitudinal strain; MW, myocardial work; GWI, global work index; GCW, global constructive work; GWW, global wasted work; GWE, global work efficiency.

Receiver operator characteristics (ROC) curve analysis indicated similar results that were observed in direct comparisons of controls and HCM subgroups of increasing LVOT gradients (Table 7 and Figure 3). The area under the curve (AUC) for GLS increased with increasing LVOT gradient categories (from 0.873 to 0.936) and were significantly different to that of controls in every HCM subgroup. On the contrary, AUC values were not different to that of controls in the obstructive HCM categories regarding GWI and GCW suggesting diminishing the discriminatory power of GWI and GCW (supporting the same observation that there is no difference between controls and oHCM patients regarding GWI or GCW). Conversely, gradual increase of AUC for GWW and GWE was observed with rising LVOT gradients (GWW: from 0.810 to 0.943), GWE: from 0.902 to 0.949) and AUC values were significantly different to that of controls in every HCM subgroup.

**Table 7:** Receiver operating characteristic (ROC) curve analysis of GLS and MW parameters between controls and different HCM subgroups of increasing LVOT gradients.

Variable	Total HCM (*n* = 118)	nHCM, <10 (*n* = 46)	nHCM, <30 (*n* = 68)	oHCM, >30 (*n* = 50)	oHCM, >50 (*n* = 40)	oHCM, >70 (*n* = 28)
	cut off values:	cut off values:	cut off values:	cut off values:	cut off values:	cut off values:
	GLS: >−17.1	GLS: >−18.5	GLS: >−17.1	GLS: >−17.1	GLS: >−17.1	GLS: >−17.1
	GWI: ≤1,688	GWI: ≤1,688	GWI: ≤1,688	GWI: >2,227	GWI: >2,227	GWI: >2,227
	GCW: ≤2,207	GCW: ≤2,207	GCW: ≤2,207	GCW: >2,749	GCW: >2,749	GCW: >2,749
	GWW: >140	GWW: >140	GWW: >140	GWW: >215	GWW: >140	GWW: >215
	GWE: ≤92	GWE: ≤92	GWE: ≤92	GWE: ≤89	GWE: ≤91	GWE: ≤91
	**AUC** **±** **SE**	**AUC** **±** **SE**	**AUC** **±** **SE**	**AUC** **±** **SE**	**AUC** **±** **SE**	**AUC** **±** **SE**
GLS (%)	**0.873** **±** 0.028****	**0.889** **±** 0.036****	**0.868** **±** 0.035****	**0.889** **±** 0.036****	**0.892** **±** 0.039****	**0.936** **±** 0.030****
GWI (mmHg%)	**0.679** **±** 0.042****	**0.844** **±** 0.047****	**0.826** **±** 0.042****	0.518 ± 0.064	0.572 ± 0.070	0.548 ± 0.084
GCW (mmHg%)	**0.664** **±** 0.042***	**0.864** **±** 0.043****	**0.838** **±** 0.040****	0.571 ± 0.064	0.637 ± 0.070	0.658 ± 0.083
GWW (mmHg%)	**0.810** **±** 0.041****	**0.689** **±** 0.060**	**0.742** **±** 0.052****	**0.908** **±** 0.035***	**0.917** **±** 0.036****	**0.943** **±** 0.030****
GWE (mmHg%)	**0.902** **±** 0.030****	**0.845** **±** 0.046****	**0.873** **±** 0.038****	**0.949** **±** 0.026****	**0.951** **±** 0.026****	**0.949** **±** 0.028****

GLS, global longitudinal strain; GWI, global work index; GCW, global constructive work; GWW, global wasted work; GWE, global work efficiency; nHCM, non-obstructive HCM; oHCM, obstructive HCM, <10, <30, >30, >50, >70, left ventricular outflow tract gradient in mmHg, respectively.

Values are given as the area under the curve (AUC) ± standard error (SE). Cut-off levels for the different comparisons are given. Values are considered statistically significantly different at

* *p* < 0.05,

** *p* < 0.01,

*** *p* < 0.001,

**** *p* < 0.0001. Significant differences are marked with asterisk and printed in bold.

### 3.5 Comparison of percentage of patients with normal GWI, GCW, GWW, GWE in HCM groups with different LVOT gradients with controls

Given the established normal ranges for GLS and MW parameters (26), we were able to calculate the proportion of patients with abnormal GLS and MW values in different HCM groups with increasing LVOT gradients (Table 8).

**Table 8:** Comparison of the proportion of patients with abnormal MW parameters between controls and different HCM groups of increasing LVOT gradients.

Variable	Controls (*n* = 32)	Total HCM (*n* = 118)	nHCM, < 10 (*n* = 46)	nHCM, < 30 (*n* = 68)	oHCM, > 30 (*n* = 50)	oHCM, > 50 (*n* = 40)	oHCM, > 70 (*n* = 28)
	*n* (%)	*n* (%)	*n* (%)	*n* (%)	*n* (%)	*n* (%)	*n* (%)
GLS	0 (0%)	**79 (66%)** ****	**31 (67%)** ****	**43 (64%)** ****	**34 (68%)** ****	**27 (68%)** ****	**21 (75%)** ***
GWI (mmHg%)	0 (0%)	13 (11%)	1 (2%)	2 (3%)	**10 (20%)** **	**9 (23%)** *	7 (25%)
GCW (mmHg%)	0 (0%)	**19 (16%)** *	2 (4%)	3 (5%)	**16 (32%)** ***	**15 (38%)** **	**13 (46%)** *
GWW (mmHg%)	2 (0%)	**62 (52%)** ****	**14 (30%)** *	**24 (36%)** **	**37 (74%)** ****	**31 (78%)** ***	**23 (82%)** **
GWE (mmHg%)	3 (0%)	**95 (79%)** ****	**31 (67%)** ****	**49 (73%)** ****	**44 (88%)** ****	**36 (90%)** ****	**25 (89%)** **

GLS, global longitudinal strain; GWI, global work index; GCW, global constructive work; GWW, global wasted work; GWE, global work efficiency.

Values are given as *n* (%). Values are considered statistically significantly different at

* *p* < 0.05,

** *p* < 0.01,

*** *p* < 0.001,

**** *p* < 0.0001. Significant differences compared to the control group are marked with asterisk and printed in bold.

GLS values were abnormal in approximately 64%–75% of patients across all HCM groups with increasing LVOT gradients, significantly exceeding the rate observed in controls. The proportion of patients with abnormal GWI or GCW values was low (2%–5%) and was comparable to controls in non-obstructive HCM groups. Although this percentage increased in obstructive HCM categories, it remained relatively low (GWI: 16%–25%, GCW: 24%–46%).

In contrast, the ratio of patients with abnormal GWW values was remarkably high even in non-obstructive HCM groups (30%–36%). In obstructive HCM categories, this ratio rose considerably (to 66%–82%), significantly surpassing the rate in controls across all HCM groups with increasing LVOT gradients. Similar trends were observed for GWE values, with a high proportion of patients (67%–73%) exhibiting abnormal values in non-obstructive HCM groups. This rate increased substantially in obstructive HCM categories (to 89%–95%), significantly exceeding the rate in controls.

## 4 Discussion

A substantial body of research has demonstrated a clear association between impaired regional strain and GLS with histopathological alterations, myocardial fibrosis, and myocardial performance in patients with HCM (27). Notably, studies have indicated that LV GLS may possess greater sensitivity than late gadolinium enhancement cardiac magnetic resonance imaging for detecting fibrosis through histopathological analysis. However, investigations exploring the relationship between GLS and outcomes in HCM have largely been limited to retrospective or prospective cohort studies. Although a majority of these studies have shown that diminished GLS is associated with adverse outcomes and survival in patients with non-obstructive HCM (28, 29) a definitive consensus regarding a clinically relevant GLS value that warrants changes in patient management remains elusive. It is important to emphasize that beyond segmental variations, GLS is susceptible to alterations from diverse pathologies, such as the not infrequent coexistence of coronary artery disease (CAD) (30) or myocardial bridging (31) in patients with HCM.

MW parameters can also be significantly confounded by several common cardiovascular comorbidities, primarily because they affect the LV pressure (the pressure component) or create inhomogeneity in myocardial strain (the strain component). Hypertension increases SBP, which automatically leads to a higher calculated GWI and GCW, and worsens LV hypertrophy and myocardial stiffness, which fundamentally changes the pressure-strain loop shape and myocardial energy use. MW is highly sensitive to the asynchronous contraction. Fibrotic or ischemic segments may contract and relax abnormally, significantly increasing GWW and reducing GWE. This high GWW may be a measure of mechanical discoordination due to fibrosis, rather than a global defect in myocyte contractility. In severe mitral regurgitation, the cuff-measured SBP used in the MW calculation greatly overestimates the effective afterload the heart is working against. This often leads to a spuriously high calculated MW, which can overstate the heart's true mechanical performance. Conduction abnormalities, like LBBB causes significant mechanical dyssynchrony, drastically increasing the GWW and lowering GWE. While this increase in GWW is a true reflection of the wasted mechanical energy, it primarily reflects an electrical problem (dyssynchrony) rather than an intrinsic myocyte contractility problem. Other comorbidities influencing MW include diabetes mellitus, chronic kidney disease and atrial fibrillation.

Since MW parameters are derived from GLS and LVP, the primary objective of this study was to delineate the differences between MW parameters and their constituent parameters in patients with HCM. Characterizing these differences would be essential to ascertain whether MW parameters provide incremental diagnostic or prognostic information beyond their component parameters, namely GLS, SBP, and LVOT gradient, or if changes in MW parameters are merely a reflection of changes in these individual components.

The major findings of our study can be summarized as follows:

### 4.1 GLS seems to be a very strong determinant of GWI and GCW, but a weaker determinant of GWW in HCM

Univariate correlation and multiple regression analyses indicated that GLS was a strong determinant of GWI and GCW. Although the correlation between GWE (a derived parameter of GCW and GWW) and GLS was also significant, it was weaker than that observed for GWI or GCW. Consistent with our findings, Hiemstra et al. (12) reported a high correlation between GCW and GLS (*r* = 0.85, *p* < 0.001). Conversely, the correlation between GLS and GWW was considerably weaker. Given the strong correlation between GLS and GWI/GCW, it is probable that changes in GLS would result in corresponding changes in GWI/GCW, potentially limiting the incremental discriminatory value of these parameters beyond GLS. In contrast, GWW appears to be the most independent parameter from GLS, exhibiting the weakest correlation and thus being the least likely to directly reflect changes in GLS to the same degree as GWI or GCW.

### 4.2 SBP seems to be generally a weaker determinant of MW parameters compared to GLS, and LVOT gradient is a determinant only in obstructive HCM

As for the other component parameters of MW, SBP seems to be a weaker determinant of MW parameters compared to GLS, except for GWW in non-obstructive HCM groups. The partial correlation between SBP and MW parameters was similar to that of resting LVOT peak gradients in both the total and obstructive HCM groups. Within the nHCM group, SBP exhibited a higher partial correlation with MW parameters compared to resting LVOT gradient. This is likely due to the minimal or absent LVOT gradients in this group, making SBP the primary determinant of LVP. In contrast, for the oHCM group, partial correlations between MW parameters, SBP, and resting LVOT gradients were comparable (although not always statistically significant), reflecting the closer proximity of LVOT gradient values to SBP values in this patient population.

### 4.3 Among MW parameters, GWI and GCW are very closely related

There is a strong correlation between GWI and GCW. Similarly, a strong correlation was observed between GWW and GWE. Therefore, changes in GCW are expected to be likely paralleled by corresponding changes in GWI, and an analogous relationship may exist between GWW and GWE.

### 4.4 With increasing LVOT gradients GWI and GCW may become “pseudonormal”, while GWW exhibits gradual increase

The key finding of this study is that with increasing LVOT gradients, GWI and GCW exhibit “pseudonormalization,” with a diminishing difference compared to controls. This is primarily attributed to the counteracting effects of a nominal decrease in GLS and a nominal increase in resting LVOT gradient. Conversely, GWW values gradually increase with rising LVOT gradients, and the difference compared to controls remains significant across all HCM subgroups. GWE displays a similar trend, although the magnitude of abnormality is relatively small. This phenomenon is consistent with the findings of Zhao et al. (15), who compared patients with nHCM, LV hypertrophy (LVH) due to hypertension, and normal controls. They observed that while GLS was reduced in both the nHCM and hypertensive groups, GWI and GCW decreased only in the nHCM group and increased in the hypertensive group, where the elevated SBP compensated for the lower GLS. In contrast, GWW values demonstrated a progressive increase across the groups. The same phenomenon was observed using ROC curve analysis as it showed that AUC values were not different to that of controls in the obstructive HCM categories regarding GWI and GCW suggesting diminishing the discriminatory power of GWI and GCW. On the contrary, gradual increase of AUC for GWW and GWE was observed with rising LVOT gradients and AUC values were significantly different to that of controls in every HCM subgroup.

### 4.5 The proportion of HCM patients with abnormal GWW or GWE seems to be much higher than that of GWI or GCW

A similar trend was observed regarding the proportion of HCM patients with abnormal MW values. The ratio of patients with abnormal GWI or GCW values remained low across increasing LVOT gradients. Conversely, the proportion of patients with abnormal GWW values was notably high, even in non-obstructive HCM groups, and increased substantially (to 66%–82%) in patients with higher levels of obstruction. GWE values exhibited a similar pattern.

These observations seem to be important comparing different patient groups or when interpreting changes in MW parameters following a specific intervention. Recently, we reported changes of MW parameters in patients treated with the direct myosin inhibitor mavacamten (32). We demonstrated that, beyond gradient reduction, MW parameters also decreased rapidly and significantly. As GLS remained unchanged, this change was primarily driven by the marked decrease in LVOT gradient. However, on the patient level, only 26% of patients had abnormal GWI (which normalized during treatment), while GWW and GWE were clearly abnormal in the majority of patients (GWW: 74%, GWE: 87%) and showed significant decreases during mavacamten treatment. Therefore, the decrease in the percentage of patients with abnormal GWW (or GWE) values reflected the beneficial effects of mavacamten on MW parameters more realistically than the nominal changes in GWI or GCW alone (or changes in the proportion of patients with abnormal GWI or GCW values, as most of the patients had normal GWI or GCW values).

The findings also give some perspective on the discriminative power of MW parameters, as opposed to GLS. As a matter of fact, there are several reports where a higher discriminative power of MW parameters over GLS were reported. In the study by Hijmstra et al. (12), patients with GCW >1,730 mmHg% experienced better event-free survival and GCW had the largest AUC of 0.78. However, GLS (AUC: 0.74) and GWI (AUC: 0.77) showed a similarly good association with the end point. Goncalves et al. also reported that a GCW cut-off of ≤1,550 mmHg% was associated with significant myocardial fibrosis with a sensitivity of 91% and a specificity of 84%, while the best cut-off for GLS (>−15%) had a sensitivity of 67% and a specificity of 76% (23). However, odds ratios were fairly similar (GCW: OR: 0.999; GLS: OR: 0.913). Brás et al. also reported that a GWI cut-off of ≤1,755 mmHg% was associated with myocardial hypoperfusion in patients with HCM, a higher sensitivity and specificity than the best cutoff for GLS (>−15.5%) (22). With regard to differentiating ATTR from HCM patients, De Gregorio reported that GWI was a better discriminator than GLS, with a cut-off value of ≤1,419 mmHg% (17). However, this is expected as both GLS and SBP were lower in patients with ATTR. GWW was also reported to be a fair predictor of composite complications (a composite of all-cause death, sudden cardiac death, myocardial infarctions, and cerebrovascular accidents) in patients with apical HCM, with a cutoff value of >186 mmHg% (16). Thus, the very complex interplay between GLS, SBP and LVOT gradient across different pathologies will determine whether MW parameters offer a higher discriminative power over GLS in patients with HCM.

## 5 Conclusions

In summary, the results of this initial technical analysis suggest that GLS is very strong determinant of GWI and GCW, but a weaker determinant of GWW in HCM, and among MW parameters, GWI and GCW are very closely related. Results also suggest that with increasing LVOT gradients GWI and GCW may become “pseudonormal”, while GWW exhibits gradual increase. Subsequently, the clinical value of MW parameters (including their incremental value over GLS) should be established by assessing their association with the morphological, clinical, and severity determinants of HCM. The final step would involve conducting a prospective investigation to determine the prognostic value of both MW parameters and GLS in predicting the outcome of HCM.

## 6 Study limitations

Several study limitations have to be mentioned. This was a single-centre analysis, and the sample size is relatively small, which confers a selection bias and limits statistical power. A further limitation, inherent to all third level cardiac centres, that HCM patients were referred from a large geographical area and patients with advanced disease or with high-risk features might have been overrepresented. In certain cases, the exact measurement of GLS may be difficult to perform due to the abnormal LV geometry inherent to the disease. This can affect inter- and intra-observer accuracy and reproducibility.

LVOT gradient is a continuous variable and, as such, affects MW parameters in a continuous manner. However, for comparative analysis, we categorized patients into non-obstructive HCM (nHCM, LVOT gradient <30 mmHg) and obstructive HCM (oHCM, LVOT gradient >30 mmHg) groups, in addition to the total HCM group. It's important to note that this threshold between these two groups is arbitrary in the context of the present study and primarily follows clinical convention. Furthermore, clinical definitions include patients with provocable gradients exceeding 30 mmHg as having obstructive HCM. To maintain consistency in our calculations, we based the nHCM and oHCM group definitions on peak resting LVOT gradients, as MW parameters are derived from these values.

## Data Availability

The original contributions presented in the study are included in the article/Supplementary Material, further inquiries can be directed to the corresponding author.
